# A hybrid deep learning and cellular automata framework with fractional derivatives for skin type and skin disease classification

**DOI:** 10.3389/fdgth.2026.1812761

**Published:** 2026-07-15

**Authors:** M. V. N. S. S. Kiranmai, C. Thanmayee Reddy, Gaddam Nikitha, Pattabiraman Venkattasubbu, Parvathi Ramasubramanian

**Affiliations:** 1 School of Computer Science and Engineering, Vellore Institute of Technology, Chennai, India; 2 Centre for Advanced Data Science, Vellore Institute of Technology, Chennai, India

**Keywords:** Caputo derivative, cellular automata, fractional derivatives, Grünwald– Letnikov derivative, Moore neighborhood, skin disease detection, skin type classification, tesellation automata

## Abstract

**Introduction:**

A hybrid deep learning and mathematical modeling approach to automatic identification of skin type and multi-class classification of skin diseases based on dermatological images are proposed in this paper. A coherent mechanism to combine deep features learning and texture modeling approaches mathematically is a necessity for improved skin type classification and skin diseases categorizations.

**Methods:**

We propose to combine convolutional neural networks, cellular automata and fractional-order derivatives in one technique. Beside CNN models based on transfer learning, this system makes use of cellular automata with online tessellation to characterize explicitly the dynamic evolution of skin textures. Specifically, a 2-dimensional Moore neighborhood cellular automata based on both totalistic and outer-totalistic rules to illustrate the local relations of neighboring pixels, the smooth aspect of skin textures, and the oily/dry distribution and pore density, which are indicators for characterizing dry, normal, and oily skin type. The models of automata characterize skin by regions and make the texture analysis stable by iterating over tessellated images. In feature extraction part, fractional-order derivatives are used for better sensitivity to edge continuity and detailed texture variations. Many types of fractional-order derivatives can be derived but the Grnwald-Letnikov and the Caputo fractional derivatives were selected for their capability to be used with discrete image grids and long-range relationship modeling. They enable better detection of delicate texture patterns that can be otherwise mistaken by traditional CNN models through an improved multiscale representation. The cellular automata-based and fractional features are combined with deep features taken from fine-tuned ResNet topologies, yielding a robust hybrid representation.

**Results & Discussion:**

The suggested method significantly reduces the misclassification of normal skin and increases skin type classification accuracy by approximately 1.2 percentage points, according to experimental evaluation on publicly available benchmark skin datasets. The integrated model achieved an accuracy of 92.8%, sensitivity of 91.4%, and F1-score of 91.7% for skin disease classification of five common dermatological conditions, while achieving an accuracy of 92.4%, sensitivity of 91.1%, and F1-score of 91.4% for skin type classification. The proposed framework therefore offers an effective and scalable approach for intelligent dermatological assessment and skin image analysis.

## 1 Introduction

Skin is the largest and most visible organ of the human body, and its condition often reflects both dermatological health and overall well-being. Preventive healthcare, cosmetic suggestions, and clinical decision support depend heavily on accurate skin type identification (dry, normal, or oily) and early detection of skin diseases. Skin analysis has always relied on visual inspection by dermatologists or cosmetic specialists, which is subjective, time consuming, and reliant on the availability of experts.Due to the demand of skin diagnosis convenience and automatic [1, 2], computer-aided skin analysis systems has become an area of much concern in recent years.

Medical image analysis has been transformed by advances in deep learning, especially convolutional neural networks (CNNs), which allow for high-accuracy classification and automated feature extraction. Several studies have established the effectiveness of CNN-based models for skin lesion and skin disease classification, often achieving performance equivalent to or above that of expert dermatologists [2, 3]. Particularly in situations with little labelled data, transfer learning using deep residual networks like ResNet has further increased classification accuracy [4, 5]. These methods have been extended to multi-class skin disease classification by the recently proposed approaches that take into consideration a broad set of dermatological diseases [6, 7]. While, most of deep learning methods, used today in this field, focus only on classifying the diseases; completely disregarding the task of classifying the type of skin explicitly where the precise differentiation of fine texture and surface characteristics is required. One of the inherent deficiencies in the current CNN based techniques lies in their black-box nature and ability to not represent the local spatial interactions and the texture changes clearly in the skin images. Especially the distinguishing of the skin type significantly relies on the microtexture like the distribution of the pores, oiliness of the skin and its smoothness that might not be perfectly learned by deep features. Mathematically based model highlighting local neighborhood interactions can be used as valuable complementary features to the deep features.

By simple local neighborhood interaction, cell automaton (CA) is a simple rule-based, discrete model which can be used to emulate complex spatial patterns. Two-dimensional CA has already demonstrated successes on several image processing tasks such as edge detection, noise removal, and texture segmentation [8, 9]. In the medical imaging field, CA has showed its ability on melanoma detection and skin area analysis in the neighborhood level texture movement [10, 11]. The model’s interpretability and success has not frequently been combined with deep learning model in skin type and disease classification.

At the same time, fractional calculus has proved to be a valuable mathematical tool to model and enhance images with multiscale textures. Because fractional-order derivatives may model non-local dependencies and preserve precise structural details, they are especially well-suited for medical images [12, 13]. Fractional differential operators improve texture sensitivity and edge continuity in skin lesion images, which improves classification performance, according to earlier research [14, 15]. While cellular automata, fractional calculus, and deep learning have been successfully applied independently to image processing and medical imaging, there has been little research combining all three methods for analysis of skin images. For that reason, an integrated approach that uses the complementary strengths of each method will provide researchers with a more accurate and interpretable analysis of the skin.

Motivated by these discoveries, the presented study suggests a new combined scheme of skin-type automatic recognition and skin-disease multi-classification automatic identification by means of deep learning, tessellation-based cellular automata and fractional order derivatives. Justification for this combination of different schemes stems from their compatible functionality that the individual three techniques individually offers distinctive contribution to the analysis task: Feature extraction of high-level semantic and geometrical information from the dermato images by using deep learning. Modelling of local texture evolution and local texture interaction between adjacent pixels by means of interpretable mechanism of cellular automata. Refinement of the texture fine details and contour smoothness of texture fine objects, by revealing large-scale spatial relation which is hard to be handled by classic image processing by applying fractional order derivatives.

This integrated approach makes use of fractional differential processing in the pre-processing and enhancement phase prior to extracting the features using the CNN to achieve better textures. Subsequently, the fine-tuned residual network is applied to the augmented images for the extraction of deep semantics, and cellular automata technique is employed to extract another set of texture descriptors. With the above methods, the proposed algorithm could increase classification accuracy and stability. The feasibility can be shown and it is implemented on a dermatological dataset. The experimental result illustrates the approach has good performance to serve as a dermatological intelligent decision support system.

## 2 Literature review

Recent accomplishments in automated skin analysis was mostly based on the use of deep learning techniques, particularly on Convolutional Neural Networks (CNN). The ability of CNN to classify skin images was confirmed by Esteva et al., whose deep neural network performs comparably to dermatologists at skin cancer detection [2]. Based on dermoscopic images, Codella et al. Focused on CNN ensemble based approaches that highlight the benefits gained from combining multiple models [3]. Using deep CNNs, Abbasi et al. introduced a multi-class skin cancer classification framework and reported significant gains in classification accuracy [6]. A systematic review by Wang et al. emphasised that transfer learning and residual networks considerably boost performance, especially when training data is restricted [4]. The superiority of deeper architectures in capturing complicated lesion characteristics was demonstrated by Hosny and Kassem’s refinement of residual deep convolutional networks for skin lesion classification [5]. In order to achieve better generalisation, Alruwaili and Mohamed used EfficientNet and ResNet models for the classification of multiclass skin diseases [7]. Deep feature representations perform better in skin disease identification than typical handmade features, according to a thorough evaluation by Bhatti et al. [16].

Despite these developments, there is still a research gap in fine-grained texture modelling for skin analysis since specific skin type classification (dry, normal, and oily) has not gotten much attention.

A significant quantity of standardised dermatoscopic images was recently proposed in order to cover the absence of freely accessible images that could enable accurate analysis of skin lesions [17]. This dataset offers a broad series of images that embody all skin lesion images that occur in daily life. Skin lesions are a class of very common skin problems accompanied by pigmentation. The overall quantity and variety of images in the dataset have been particularly high, enabling a broad variety of clinical sources that cover different kinds of skin lesions. This dataset was presented in order to facilitate various machine learning and deep learning activities, and its appearance has significantly contributed towards developing and evaluating automated skin lesion classification systems more equitably.

Cellular automata (CA) provides a framework of simulated emergence of texture and local spatial interactions. In CA, a mathematically grounded theory of cellular automata was developed by Wolfram [8, 18]. Nandi et al. studied two-dimensional CA applications in image processing, demonstrating effectiveness in edge recognition and texture analysis [9]. While Naskar and Chatterjee suggested CA-based edge detection techniques, Das and Chanda used CA for noise reduction [19, 20]. For the best edge identification, Ahmed and El-Sayed expanded CA models with bigger neighbourhood structures [21]. Luna-Benoso et al. demonstrated the potential of CA in dermatological analysis by using CA classifiers for melanoma identification in medical imaging [10]. CA was further studied by Bandini and Manzoni as a flexible image processing tool [11].

Fractional calculus has emerged as a valuable tool for image improvement due to its ability to describe non-local and multiscale interdependence. Podlubny and Ortigueira offered essential formulations of fractional differential equations [12, 13]. Texture and edge continuity are enhanced using fractional differential masks [14]. In order to detect melanoma, Zhang et al. used fractional differentiation, which resulted in better boundary delineation [15]. Fractional differential equations have been shown to be useful for improving medical images by Chen et al. [22]. Robust spatial modelling in discrete image domains is further made possible by Caputo’s fractional derivative formulation [23].

Promising outcomes have been demonstrated by hybrid approaches that combine mathematical modelling and deep learning. In order to classify skin lesions, Jamal et al. presented TopoResNet, which combines topological characteristics with CNNs [24]. A system that utilized deep neural networks for multi-disease classification of skin was proposed by Ali et al. [25]. Inspired by this work, this paper proposes a hybrid system integrating cellular automata, fractional calculus and deep learning approaches, aiming to reach interpretable and robust classification of skin type and skin disease. An automatic detection and classification of skin lesion using K-means and LAB color space segmentation approaches were applied by Jabber et al. [26]. This technique involved using image segmentation along with deep learning methods in order to detect lesions and perform classification. The results showed that combining the two techniques can be effective in dermatological image analysis.

Table 1 gives a comparative review of the proposed framework with representative current studies in skin image analysis. In the past there have been studies evaluating the effectiveness of cellular automata, fractional modelling, and deep learning when individually implemented on skin-analysis tasks, These studies provide valuable insights into feature extraction, data augmentation, and modelling. Our approach based on the above-mentioned methods, utilizes methods that complement one another in the hybrid model for achieving performance similar to existing methods, and also maintaining the interpretability of the results on skin type and disease classification.

**Table 1:** Comparison of proposed work with recent studies.

Study	Method	Features	Remarks
Esteva et al. [2]	CNN	Deep features	Landmark DL work; lacks texture modeling
Codella et al. [3]	Ensemble CNN	CNN ensembles	Robust fusion; low interpretability
Abbasi et al. [6]	Deep CNN	CNN features	Multi-class focus; no skin type analysis
Luna-Benoso et al. [10]	Cellular Automata	CA rules	CA-only; no deep features
Zhang et al. [15]	Fractional + CNN	Fractional texture	Improves edges; no CA
Jamal et al. [24]	TopoResNet	Topological + CNN	Hybrid; not texture-specific
Proposed work	CNN + CA + Fractional	Hybrid features	Skin type + disease, interpretable

## 3 Proposed work

**Figure 1:**
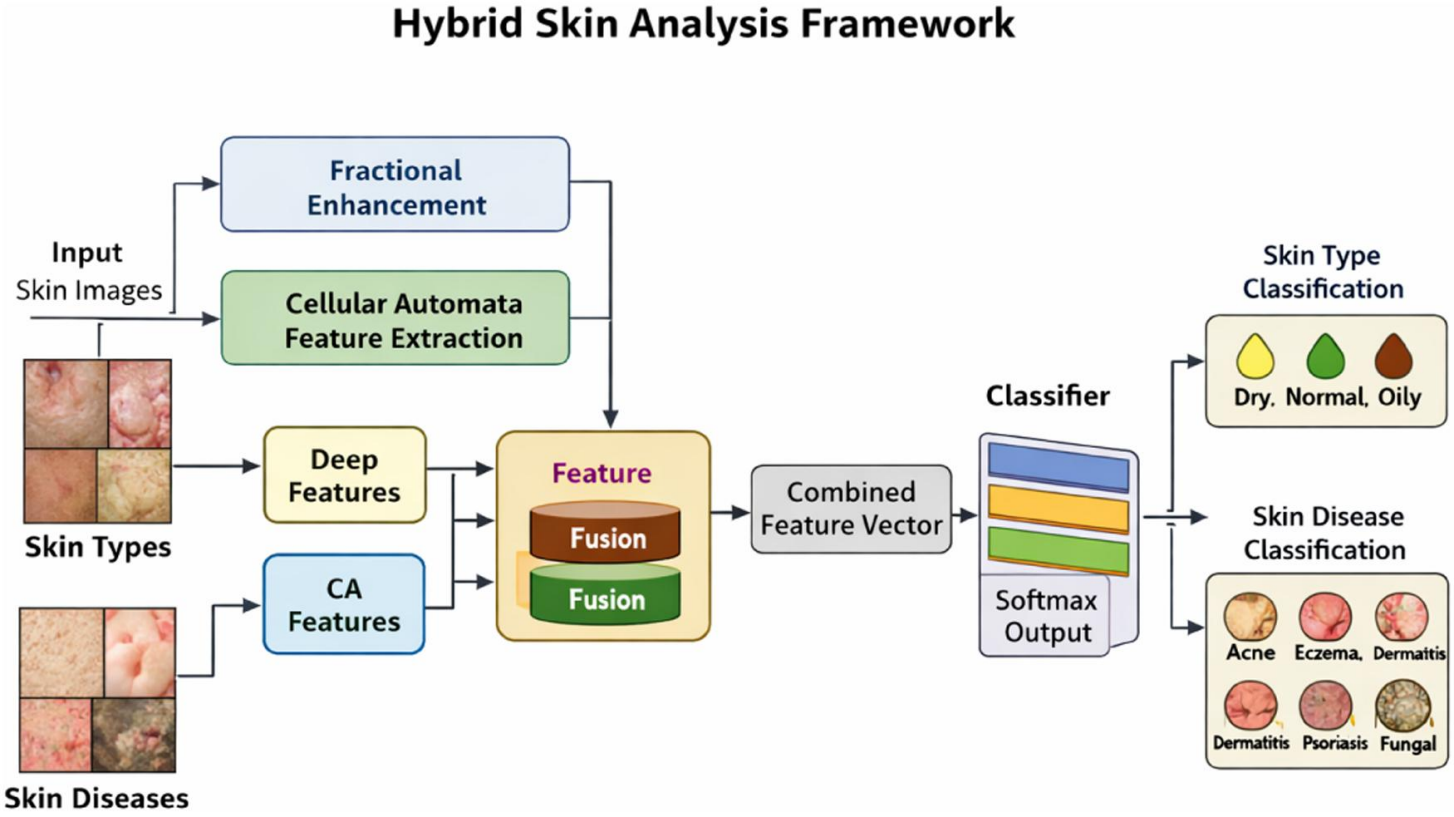
System architecture.

This proposed work outlines a hybrid and intuitive system for the automatic detection of skin types and the classification of non-cancerous skin diseases. Our aim is to combine mathematical modelling with deep learning so as to accurately capture both local texture features and global dermatological features. The entire system is designed as a modular pipeline in which skin image inputs are progressively processed until a classification is reached. While the technical specifics of each step are detailed in the parts that follow, this section presents a simplified perspective of the workflow. The entire system architecture is depicted in Figure 1, It serves as a visual reference for the flow of procedures outlined below.

The image acquisition process is the first step in this proposed work; the dermatological images were acquired both from real-world and available datasets. All the acquired images were subjected to same preprocessing module in order to make the image resolution uniform and remove the noise and enhancement from images and prepare for the subsequent processes. It also uses Fractional Order enhancement to enhance the surface and texture features of the dermatological images, which allows system to achieve contrast of fine structures like pore, small fissures.

Following the image enhancement stage, the image is passed to the cellular automata module where it generates semantically relevant texture descriptors, using tessellation to distinguish skin types and learn neighborhood relationships on the small image regions. At the same time, the image is passed through the deep convolutional neural networks to extract relevant high-level semantic features for skin type classification. Features extracted by the two techniques are then combined using a hybrid feature-fusion strategy.

Lastly, the joint feature representation is fed into the classification layers which generate the two outputs: the prediction of skin type and the detection of the relevant healthy skin disease correspondence. Finally the class labels and their confidence score can be generated. The output of the system is the class label and its confidence value, which provides useful decision support for skin assessment. In summary, this system effectively combine deep learning with mathematical modeling to produce skin analysis that is accurate, explainable, and scalable.

The remaining sections describe each module of the proposed system in detail. Section 4 outlines the image preprocessing and fractional enhancement procedures. The modelling of cellular automata based on tessellation is shown in Section 5. The deep learning architecture for feature extraction is described in Section 6. Section 7 discusses the feature fusion and classification methodologies, while Section 9 gives that experimental findings and analysis, followed by conclusions in the concluding section.

## 4 Image preprocessing and enhancement

Variations in illumination, noise artefacts, and non-standard resolutions are common problems with dermatological photographs obtained from clinical and internet repositories. If employed directly for model training, such discrepancies could seriously impair classification performance. Therefore, prior to feature extraction, a systematic preprocessing and enhancement pipeline is used to enhance visual quality, stabilise contrast, and highlight minor skin texture characteristics. The basic phases of preprocessing are noise removal, intensity normalisation, resizing, data augmentation, and fractional-order based enhancement.

### 4.1 MONAI-based medical image preprocessing

Modern dermatological image analysis benefits from pretreatment processes that are specifically intended for medical imaging data. The Medical Open Network for AI (MONAI) framework is used in this work to improve image preparation robustness and standardisation. MONAI ensures the robustness of downstream learning models by providing a variety of domain-specific adaptations to ensure consistency over multiple medical data sets.

The input skin images are converted to a channel-first format for seamless compatibility with deep learning networks. The pixel values of the images are normalized to an appropriate range by performing intensity normalization using MONAI library to account for lighting conditions variations that may exist as a result of different image acquisition setups. This is very important in dermatological imaging aslighting variations can cover up minor texture characteristics attributed to skin type or condition.

We use the MONAI transformations in order to make these spatial normalisations such as padding and scaling keeping the aspect ratios in order to maintain a fixed resolution at the input. While standard scaling algorithms alter the aspect ratio, the MONAI scaling can maintain the spatial relationships, which are necessary to the analysis of skin textures and boundaries of the lesions. These methods are combinable with the described augmentation techniques and with standard noise filters.

The fractional-order images are enhanced with the steady and controlled input from the MONAI preprocessing pipeline. As fractional derivatives highlight fine texture and edge continuity, this normalization technique through MONAI means that such improvements are also applied consistently across the data. The benefit in training is better texture detail and better generalizability.

### 4.2 Noise removal

Images of the skin often have sensor noise and environmental artefacts, along with compression artifacts. The use of a combination of median and Gaussian filters is implemented to minimize these changes whilst preserving diagnostic edges.

Given an image I(x,y), the median filter output $\hat{I}(x,y)$ is defined as:

$$\hat{I}(x,y)=\text{median}\{I(i,j)\},\;(i,j)\in N(x,y),$$ (1)

where N(x,y) represents the local neighborhood window centered at (x,y). As shown in Equation 1, median filtering replaces each pixel value with the median of its local neighborhood. This process effectively suppresses impulsive noise while preserving important image structures.

Subsequently, Gaussian filtering is used to suppress high-frequency noise components:

$$G(x,y)=\frac{1}{2\pi \sigma^{2}}e^{-\frac{x^{2}+y^{2}}{2\sigma^{2}}},$$ (2)

and the denoised image becomes:

$$I_{f}(x,y)=I(x,y)*G(x,y),$$ (3)

where ∗ denotes convolution. As defined in Equation 2 and applied through convolution in Equation 3, Gaussian filtering suppresses high-frequency noise while preserving the overall structural integrity of dermatological images.

### 4.3 Normalization

The constant intensity distribution over the varying images is achieved by normalization. The pixel intensities are scaled to the range [0,1] by using min–max normalization,defined in Equation 4.

$$I_{n}(x,y)=\frac{I_{f}(x,y)-I_{\min}}{I_{\max}-I_{\min}},$$ (4)

Here, $I_{\min}$ and $I_{\max}$ represent the minimum and maximum global intensities respectively. This normalization process helps in eliminating the influence of illumination variation, steady the contrast and allow for rapid and stable convergence.

### 4.4 Resizing

Normalized images are resized to a fixed spatial resolution of 224×224 pixels to ensure compatibility with deep learning architectures. The resizing operation is defined in Equation 5.

$$I_{r}=R(I_{n},224,224),$$ (5)

Here, R(⋅) denotes the resizing operator. By fixing the input size, we have uniform sizes of feature maps under convolution operations and network training would be more stable.

### 4.5 Data augmentation

For training phase, to combat overfitting and enhance model generalization, geometric and photometric variations are used, which involves the application of data augmentation techniques. The augmentation process is formulated in Equation 6.

$$I_{a}=T(I_{r}),$$ (6)

Here, T(⋅) denotes a stochastic transformation operator that includes horizontal flipping, rotation, random cropping, and mild brightness perturbations. These transformations simulate realistic image acquisition conditions and enhance dataset diversity.

### 4.6 Fractional-order image enhancement

Conventional integer-order operators have some difficulty in preserving both sharp edges and smooth surfaces at the same time. Fraction order derivatives are utilized to improve rich skin textures and remove noise to overcome these drawbacks. Because fractional order derivatives can model non-local relations between points of skin, they can realize multi-scale enhancement.

#### 4.6.1 Grünwald–Letnikov fractional derivative

The Grünwald–Letnikov (GL) formula gives a definition of a fractional order derivative for a discrete signal and a way to model non local spatial relations. The GL fractional derivative is expressed in Equation 7.

DGLαf(x)=limh→01hα∑k=0⌊x/h⌋(−1)k((αk))f(x−kh), (7)

Here, α∈(0,1) denotes the fractional order controlling the degree of differentiation. When applied to image gradients, this formulation enhances fine-scale texture patterns such as pores and micro-wrinkles while preserving structural continuity.

#### 4.6.2 Caputo fractional derivative

The Caputo fractional derivative is defined as

$$D_{C}^{\alpha }f(x)=\frac{1}{\Gamma (1-\alpha )}\int_{0}^{x}\frac{\;f^{\prime }(t)}{(x-t{)}^{\alpha }}\;dt,$$ (8)

where Γ(⋅) denotes the Gamma function and α∈(0,1) represents the fractional order. The formulation in Equation 8 produces smoother intensity transitions at region boundaries, thereby preserving lesion contours while minimizing enhancement-induced distortion.

#### 4.6.3 Role in texture enhancement

Fractional derivatives offer a flexible trade-off between edge emphasis and smoothing. Higher values of α tend to emphasize small textural patterns; lower values result in a smoother image in global sense. This is useful for skin analysis because discriminative cues for different skin types are often at the micro-texture level instead of at an abrupt edge. Proposed method improves traits for different skin types, boundary continuity and pore patterns using fractional-order operators as preprocessing stage.

The combined procedure for preprocessing and enhancement of skin images brings the great improvements in visual quality and discriminatory power of the images, which sets up a good foundation for subsequent feature extraction and classification.

## 5 Tessellation-based cellular automata modeling

Cellular automata (CA) is a discrete, grid based computer simulation model which globally observed images arise from local operations ruled by few updating rules. We used cellular automata to make explicit account for the texture change on images on skin surface in order to develop a human understandable skin pore distribution, skin oil density and skin surface smoothness. cellular automata rules define the neighbourhood relation on next states as well as on subsequent states instead of on local convolution filters, which will give an intuitive model of how texture local change goes all over the skin surface.

### 5.1 Tessellation grid generation

Let I(x,y) denote the preprocessed image. The image is partitioned into a two-dimensional grid of uniformly tessellated cells,

$$G=\{c_{ij}|i=1\ldots M,\;j=1\ldots N\},$$ (9)

where each cell $c_{ij}$ corresponds to a small local image patch.

Each cell maintains a state value $s_{ij}^{\left(t\right)}$ at iteration t, initialized based on local pixel characteristics such as intensity, variance, or gradient magnitude:

$$s_{ij}^{\left(0\right)}=\phi (c_{ij}),$$ (10)

where ϕ(⋅) denotes the state initialization operator. The tessellation process defined in Equations 9 and 10 enables skin texture to be modeled as an organized spatial system, rather than as independent pixel intensities.

### 5.2 Moore neighborhood modeling

For each cell $c_{ij}$, interactions are defined over a Moore neighborhood that includes the cell itself and its eight surrounding neighbors,

$$N_{ij}=\{c_{\;p,q}||p-i|\leq 1,\;|q-j|\leq 1\}.$$ (11)

This neighbourhood structure takes into account both horizontal and diagonal connections which permits the modeling of irregular clusters of pores and irregular surfaces usually observed in dermatological images. The neighborhood definition in Equation 11 allows each cell to update its state based on the collective influence of adjacent regions.

### 5.3 Totalistic update rules

In a totalistic cellular automaton, the next state of a cell is determined exclusively by the sum of the states of its neighbors. The totalistic update rule is given by

$$s_{ij}^{\left(t+1\right)}=f\left(\sum_{(p,q)\in N_{ij}}s_{\;p,q}^{\left(t\right)}\right),$$ (12)

where f(⋅) denotes a nonlinear activation function that applies scaling or thresholding to the neighborhood sum. The update mechanism defined in Equation 12 emphasizes homogeneous regional textures, such as uniformly dry or oil-rich areas, while suppressing local irregularities.

### 5.4 Outer-totalistic update rules

Outer-totalistic rules build on totalistic rules to include both the center cell’s state and the sum of its neighbors’ influences. The update rule is given by

$$s_{ij}^{\left(t+1\right)}=f\left(\alpha s_{ij}^{\left(t\right)}+\beta \sum_{\left(p,q)\in N_{ij}\setminus (i,j\right)}s_{\;p,q}^{\left(t\right)}\right),$$ (13)

where α and β are tunable parameters that control self-influence and neighborhood influence, respectively. The formulation in Equation 13 enables a balanced evolution of cell states by preserving individual texture consistency while maintaining spatial continuity across surrounding regions.

### 5.5 Iterative evolution process

The cellular automata system evolves over a fixed number of iterations to allow spatial texture patterns to emerge,

$$G^{\left(t+1\right)}=F(G^{\left(t\right)}),\;t=0,\ldots ,T-1,$$ (14)

where F denotes the global update operator derived from local neighborhood transition rules. The iterative process defined in Equation 14 enables small local variations to propagate across the grid, leading to stable texture formations: dry skin typically evolves into coarse-grained patches, oily skin forms smooth and highly connected clusters, and normal skin converges to balanced intermediate patterns. The entire algorithmic framework for skin texture classification based on cellular automata is outlined in the Algorithm 1, where the image is divided into local cells, updated using Moore neighbors' rules, and converted into texture features.

Algorithm 1 **Cellular Automata-based Skin Texture Classification.**

**Require:** Input image *I*, number of iterations T

**Ensure:** Predicted class label y

1: Divide *I* into tessellated grid G

2: Initialize cell states $S_{i,j}^{\left(0\right)}$ from local intensity statistics

3: **for** t=0 to T−1 **do**

4:  **for** each cell (i,j) inG **do**

5:   Determine Moore neighborhood $N_{i,j}$

6:   Compute neighborhood sum $H=\sum_{(p,q)\in N_{i,j}}S_{\;p,q}^{\left(t\right)}$

7:   Update state:

             $S_{i,j}^{\left(t+1\right)}=f(H)$

8:  **end** **for**

9: **end** **for**

10: Extract texture feature vector $f_{CA}$

11: $y\leftarrow Classifier(f_{CA})$

12: **return** y

### 5.6 Texture feature extraction

Upon convergence of the cellular automata, feature extraction using texture descriptors are extracted from the resultant grid,

v=[mean(s),variance(s),entropy(s),contrast(s)], (15)

where s denotes the final grid state. The feature vector defined in Equation 15 is further enriched with additional morphological texture measures, including neighborhood correlation, roughness index, and cluster compactness, yielding an interpretable representation of skin texture patterns.

In conclusion, a cellular automaton approach to skin texture modelling using tessellation offers a mathematically principled framework connecting individual pixels to skin texture semantics in parallel with the deep learning network, thereby enriching the hybrid approach in diagnostic utility.

## 6 Deep learning model

The paper proposed the utilization of deep learning for the extraction of hierarchically semantically representative features to characterize both structural and chromatic characteristic features of dermatological patterns. The CNN provides the high-level features, such as boundary shape, chromaticity distribution, abnormalities of shapes, and cell automaton model provides the low-level features, such as local correlation between neighboring pixel values of the textures. This section is going to present the structure of deep learning network, transfer learning approaches and feature extraction procedure.

### 6.1 Residual network architecture

Residual Networks (ResNet) are adopted as the backbone architecture due to their ability to train very deep models without performance degradation. A ResNet block introduces identity-based skip connections, defined as

y=F(x,W)+x, (16)

where x denotes the input feature map, F(⋅) represents the residual mapping learned by the convolutional layers, and W corresponds to the trainable parameters. The formulation in Equation 16 facilitates efficient gradient propagation, mitigating vanishing gradient issues and enabling stable optimization of deep networks.

In this work, two variants are adopted:

- ResNet-50 for skin type classification,
- ResNet-152 for multi-class skin disease recognition.

Both models consist of layered convolutional, batch normalisation, and ReLU activation layers followed by global average pooling and fully connected classification heads.

### 6.2 Transfer learning

The medical imaging datasets may be limited for training deep networks from scratch. In this scenario, the transfer learning approach can be applied effectively. ImageNet weights are utilized to initialize the network weights, allowing early convolutional layers to learn general features such as edges and textures, while subsequent layers are tuned for specific dermatological patterns. During fine-tuning, model optimization is achieved by minimizing the classification loss defined in Equation 17.

$$L(\theta )=-\sum_{i=1}^{N}\sum_{k=1}^{C}y_{ik}\log({\hat{y}}_{ik}).$$ (17)

Here, θ denotes the set of trainable parameters, N represents the number of training samples, and C is the number of output classes. The variable $y_{ik}$ corresponds to the ground-truth label, while ${\hat{y}}_{ik}$ denotes the predicted class probability obtained through the Softmax function.

### 6.3 Feature extraction process

For each input image, the pretrained network extracts hierarchical feature maps of increasing abstraction. A compact deep feature representation is obtained using global average pooling, as defined in Equation 18.

$$f_{DL}=\text{GAP}(F).$$ (18)

Here, F denotes the output of the final convolutional block. The resulting deep feature vector captures lesion shape, intensity gradients, and color variation patterns, and is subsequently normalized before being forwarded to the feature fusion stage.

Table 2 summarises the classification accuracy achieved by different ResNet variants across multiple modelling methodologies. Combining fractional modelling and cellular automata components consistently improves the performance of both ResNet-50 and ResNet-152. The hybrid framework benefits from richer ResNet representations, resulting in improved accuracy compared to individual model setups. These data show that integrating architectural depth with complementary modelling methodologies helps to improved classification performance.

**Table 2:** Accuracy comparison of ResNet variants across different models.

ResNet variant	CNN	CA	Fractional + CNN	Hybrid (CNN + CA + Fractional CNN)
ResNet-50	0.905	0.824	0.914	0.936
ResNet-152	0.912	0.831	0.921	**0.944**

Bold values are representing the performance of hybrid algorithms.

### 6.4 Vision transformer (ViT) for skin image representation

The efficiency of attention-based global feature modelling for skin image classification is assessed in this work using Vision Transformer (ViT). ViT splits the input skin image into fixed-size patches and uses self-attention methods to process them, in contrast to convolutional networks that concentrate on local receptive fields. Long-range dependencies including global texture consistency, lesion spread, and colour fluctuation throughout the skin’s surface can thus be captured by the model. The CNN-based method employed in the suggested framework is experimentally contrasted with the performance of ViT. In order to evaluate complementary benefits, a hybrid design that combines ViT representations with cellular automata features is also assessed. Table 3 provides a comparative analysis of baseline models and the hybrid framework improved using ViT. When used alone, individual methods such as CNN, cellular automata, and fractional-enhanced CNN show competitive performance. The hybrid model benefits from the integration of complementary components, producing higher accuracy and F1-score. These findings suggest that integrating transformer-based representations, deep learning, and mathematical modelling can improve classification performance even more. The suggested framework utilizes only the integration of CNNs, cellular automata, and fractional order derivatives. Vision Transformer (ViT) was considered as an additional benchmark to examine the performance of transformer representations against the suggested hybrid framework.

**Table 3:** Performance comparison of baseline models and proposed hybrid model with ViT enhancement.

Model	Accuracy	F1-Score
CNN	0.912	0.908
Cellular Automata (CA)	0.824	0.818
Fractional + CNN	0.918	0.914
**Hybrid (CNN + CA + Fractional CNN + ViT)**	**0.932**	**0.927**

Bold values are representing the performance of hybrid algorithms.

### 6.5 Why deep learning complements cellular automata

Local neighbourhood changes are modelled by cellular automata, but they are not very good at global structural reasoning. On the other hand, deep CNNs do not explicitly encode local interaction rules, yet they are excellent at capturing intricate spatial hierarchies. Combining both brings complementary strengths:

- CA extracts interpretable texture descriptors,
- CNNs capture high-level patterns and semantic boundaries,
- Fusion yields rich hybrid representations enabling improved classification.

To integrate complementary information from deep learning and cellular automata modeling, deep features and CA-based texture features are combined using feature concatenation, as defined in Equation 19.

$$f_{H}=[f_{DL}\;\|\;f_{CA}].$$ (19)

Here, ‖ denotes the concatenation operator, resulting in a unified hybrid feature representation that jointly captures high-level semantic patterns and local texture characteristics for subsequent classification.

### 6.6 Task-specific adaptation

Two learning heads are defined for domain-specific categorisation tasks.

#### 6.6.1 Skin type model

Using ResNet-50, the skin type model divides input photos into three categories: dry, normal, and oily. A completely linked layer followed by Softmax activation is implemented. Training performance was optimised using Adam optimiser with tuned parameters.

#### 6.6.2 Skin disease model

ResNet-152 is used by the skin disease model to categorise photos into several non-cancerous disease groups. Stronger learning capacity for fine structural changes among lesion types is made possible by the greater depth.

## 7 Feature fusion and classification

The suggested approach combines deep convolutional networks and heterogeneous feature representations produced by cellular automata modelling. Deep learning features encode global morphological and semantic signals, whereas CA features reflect local neighbourhood interactions and micro-texture change. The algorithm can make more trustworthy conclusions by combining the data from both sources, especially when there are slight visual variations across skin states. The feature fusion mechanism and the ultimate classification procedure are explained in this section.

### 7.1 Fusion strategy

Let $f_{CA}$ denote the texture descriptor vector extracted from the tessellation-based cellular automata model, and let $f_{DL}$ represent the deep feature vector obtained from the final global average pooling layer of the ResNet network. To form a unified hybrid representation, both feature vectors are first normalized and then concatenated, as defined in Equation 20.

$$f_{H}=[\text{norm}(f_{CA})\;\|\;\text{norm}(f_{DL})].$$ (20)

Here, ‖ denotes vector concatenation. Feature normalization ensures balanced contributions from both modalities during learning, preventing dominance by any single feature group. The constructed hybrid feature space simultaneously represents both local texture patterns and more abstract lesion characteristics for classification.

### 7.2 Classifier structure

The fused hybrid feature vector is passed to a multi-layer fully connected classifier composed of dense layers, batch normalization, and dropout regularization. The classifier transforms the hybrid representation into discriminative decision boundaries, as defined in Equation 21.

$$z=\sigma \left(W_{2}\sigma (W_{1}f_{H}+b_{1})+b_{2}\right).$$ (21)

Here, $W_{1}$ and $W_{2}$ denote learnable weight matrices, while $b_{1}$ and $b_{2}$ represent bias terms. The nonlinear activation function σ(⋅) corresponds to the ReLU function. Dropout regularization is applied during training by randomly deactivating neurons, thereby reducing overfitting and improving generalization.

Two dedicated classification heads are implemented:

- Skin type head: classifies images into dry, normal, or oily skin.
- Skin disease head: predicts the corresponding non-cancerous dermatological category.

This dual-output design allows joint learning across related tasks while preserving task-specific decision optimization.

### 7.3 Softmax probability estimation

The classifier produces a probability distribution over all target classes using the Softmax function, which converts the output logits into normalized confidence scores, as defined in Equation 22.

$$P(y=k|z)=\frac{\exp(z_{k})}{\sum_{\;j=1}^{C}\exp(z_{j})}.$$ (22)

Here, $z_{k}$ denotes the logit corresponding to class k, and C represents the total number of output classes. The Softmax formulation ensures that all predicted class probabilities sum to one, enabling interpretable confidence estimation for the classification outcomes. The Softmax probability calculation and class prediction procedure can be summarized formally using the Algorithm 2, where the normalization of the logits to class probabilities takes place and the most probable class is predicted finally.

Algorithm 2 **Softmax Probability Estimation and Class Prediction.**

**Require:** Logit vector $z=[z_{1},z_{2},\ldots ,z_{C}]$

**Ensure:** Predicted class label y

1: Initialize sum_exp←0

2: **for** j=1 to C **do**

3:  $sum\_exp\leftarrow sum\_exp+\exp(z_{j})$

4: **end** **for**

5: **for** k=1 to C **do**

6:  $P[k]\leftarrow \exp(z_{k})/sum\_exp$

7: **end** **for**

8: $y\leftarrow \arg\underset{k}{\max}\;gtP[k]$

9: **return** y

### 7.4 Decision logic

The final class label is determined using maximum probability selection based on the Softmax output, as defined in Equation 23.

$$\hat{y}=\arg\max_{k}P(y=k|z).$$ (23)

According to this decision rule, the class with the greatest predicted probability will be chosen. That is to say that this rule selects the skin type or disease category with the highest chance. In addition, probability margins are analyzed to estimate prediction confidence, and cases with confidence values below a predefined threshold are flagged for manual review to enhance clinical safety and reliability.

When compared to single-modality methods, the hybrid feature fusion paradigm greatly improves classification performance. While deep learning offers strong semantic representations, cellular automata contribute interpretable textural descriptors. The combination supports possible inclusion into automated dermatological decision-support systems by producing complementing discriminative power and providing interpretable information into model decisions.

## 8 Experimental setup

### 8.1 Dataset description and data partitioning

The proposed framework was evaluated using publicly available dermatological image datasets for both skin disease classification and skin type classification tasks.

For the skin disease classification category, images were collected from the DermNet dermatological image database. In this regard, five skin conditions were selected to include in this experiment, which are Acne, Dermatitis, Eczema, Psoriasis, and Fungal Infection. The total number of images in this experimental dataset was 5,500.

In the case of skin type classification, a publicly available dry_oily_normal Skin Dataset collected from Roboflow was used. This dataset included three skin categories that are Dry Skin, Normal Skin, and Oily Skin having a total of 1,072 images.

To ensure fair and reproducible evaluation, the datasets were partitioned using a stratified 70:15:15 split, where 70% of the samples were used for training, 15% for validation, and 15% for testing. The same data partitions were used across all evaluated models.

Both the skin disease and skin type classification were performed on the following datasets which are summarized in Table 4. Public datasets were used for the two classification problems in order to make the results reproducible.

**Table 4:** Dataset summary.

Classification task	Dataset	Classes	Total images
Skin disease classification	DermNet Repository	5	5,500
Skin type classification	Roboflow dry_oily_normal Skin Dataset	3	1,072

Distribution of Images in the Classes for Skin Disease Classification is Shown in Table 5. The skin diseases dataset includes five dermatological diseases selected from DermNet database.

**Table 5:** Skin disease dataset distribution.

Disease category	Images used
Acne	1,200
Dermatitis	900
Eczema	1,500
Psoriasis	1,100
Fungal infection	800
Total	5,500

The image distribution on each category of the skin types is depicted in Table 6. The dataset consists of the three skin types: Dry Skin, Normal Skin, and Oily Skin.

**Table 6:** Skin type dataset distribution.

Skin type	Images used
Dry skin	357
Normal skin	357
Oily skin	358
Total	1,072

The proposed data splitting method for this project is provided in Table 7. The method of data splitting used was the stratified 70:15:15 for both classification tasks.

**Table 7:** Dataset partitioning strategy.

Dataset	Training (70%)	Validation (15%)	Testing (15%)
Skin disease dataset	3,850	825	825
Skin type dataset	750	161	161

### 8.2 Deep learning training configuration

The parameters involved in the implementation and training of the suggested deep learning model are outlined in Table 8. Transfer learning was adopted, which entailed loading pre-trained models of ResNets through ImageNet and thereafter fine-tuning the task-dependent layer in order to classify dermatological images. Experimentation was done via TensorFlow software using GPU-accommodating systems. The training configuration is consistent across the CNN, Fractional-CNN, and Hybrid models.

**Table 8:** Deep learning training configuration.

Parameter	Value
Learning rate	0.001
Batch size	32
Epochs	30
Optimizer	Adam
Fine-tuning strategy	ResNet pretrained on ImageNet; final layers fine-tuned
Random seed	42
Early stopping	Patience=5
Software environment	TensorFlow
Hardware environment	GPU-enabled computing environment

### 8.3 Multi-resolution input analysis

The suggested system assesses skin photos at several spatial resolutions, including 16×16, 32×32, 64×64, 128×128, and 256×256, in order to examine the impact of input resolution on skin type and skin disease classification. This experiment provides an insightful analysis of how different levels of spatial resolution influence the understanding of semantic content, the representation of textures, and classification accuracy.

### 8.4 Texture information capture

While fine-grained surface features like pores, microcracks, and small pigmentation changes are lost, coarse texture information is largely preserved at lower resolutions like 16×16 and 32×32. Lower classification accuracy results from models trained on these resolutions relying mostly on global intensity distribution rather than significant texture dynamics.

### 8.5 Balance between texture and semantic features

A balance between local texture details and global structural information is provided by intermediate resolutions, especially 64×64 and 128×128. Since neighbourhood interactions are still significant and there is enough spatial context for deep learning models, these resolutions are ideal for fractional-order augmentation and cellular automata modelling. As a result, at these scales, classification performance greatly improves.

### 8.6 High-resolution semantic understanding

Deep learning algorithms can extract richer semantic representations because high-resolution inputs, such 256×256, preserve intricate lesion borders, colour changes, and structural imperfections. Beyond a certain point, however, very high resolutions only slightly improve efficiency because they also raise computing complexity and may include redundant background information. The effects of different input image resolutions on classification performance for CNN, CA, fractional-enhanced CNN, and the suggested hybrid framework are shown in Table 9. Better feature representation at higher spatial scales is indicated by the results, which consistently demonstrate improvements in accuracy, precision, and F1-score with increasing resolution for all models. The hybrid model continuously performs better than the baseline and intermediate models among the assessed methods at all resolutions. The 256×256 resolution yields the best results, indicating the resilience and efficiency of the proposed framework. The complete hybrid classification process used in the proposed approach is given by Algorithm 3, which includes preprocessing, fractional order enhancement, cellular automata based modeling of texture, deep features extraction, feature fusion, and ultimately prediction.

**Table 9:** Effect of input image resolution on classification performance across different models.

Input res	CNN			CA			Fractional+CNN			Hybrid		
	Acc	Prec	F1	Acc	Prec	F1	Acc	Prec	F1	Acc	Prec	F1
16×16	0.821	0.815	0.812	0.768	0.761	0.758	0.832	0.826	0.823	**0.845**	**0.838**	**0.835**
32×32	0.854	0.848	0.846	0.801	0.794	0.792	0.865	0.859	0.857	**0.878**	**0.872**	**0.870**
64×64	0.892	0.887	0.885	0.834	0.828	0.826	0.904	0.898	0.896	**0.916**	**0.910**	**0.908**
128×128	0.918	0.912	0.910	0.862	0.856	0.854	0.928	0.922	0.920	**0.936**	**0.930**	**0.928**
256×256	0.921	0.914	0.912	0.868	0.861	0.859	0.931	0.925	0.923	**0.944**	**0.938**	**0.936**

Bold values are representing the performance of hybrid algorithms.

## 9 Results and discussion

The experimental results from the suggested hybrid framework are presented and examined in this section. Benchmark dermatological datasets including examples of non-cancerous skin diseases and photos of different skin types were used to evaluate the model. Each experiment was conducted three times to guarantee statistical consistency, and the arithmetic mean of the outcomes was noted. Since depending solely on one statistic does not fairly depict actual clinical behaviour, a variety of performance metrics were taken into consideration, including accuracy, precision, recall, specificity, and F1-score. Together, these measures assess the model’s accuracy in class identification, its frequency of avoiding incorrect predictions, and its ability to manage case imbalance.

The skin diseases classification tests were done using five types of skin diseases, which include Acne, Dermatitis, Eczema, Psoriasis, and Fungal Infections. The above skin diseases were chosen since they are some of the typical skin diseases that show different appearances, making it ideal to evaluate the hybrid classifier framework suggested.

### 9.1 Explainability through heatmap visualization

To determine which areas of the skin image contribute more to the prediction, heatmap visualisation is used in the suggested framework following the classification step. Gradient-based activation mapping is calculated using the final convolutional feature maps after the trained deep learning model generates a classification output. An attention map is created by propagating the gradients of the anticipated class back to these feature maps. The input skin image is superimposed with this attention map, which has been scaled to the original image resolution. The impacted skin areas that affected the model’s choice are indicated by regions with greater intensity values. The exact localisation of afflicted skin areas is made possible by the focused activations over lesion and aberrant texture regions produced by the application of fractional-order enhancement and cellular automata modelling.

Algorithm 3 **Hybrid CNN–CA–Fractional CNN Classification.**

**Require:** Image *I*, fractional order α, CA iterations *T*, CNN parameters θ

**Ensure:** Predicted class label y

1: $I_{d}\leftarrow NoiseRemoval(I)$

2: $I_{n}\leftarrow Normalize(I_{d})$

3: **for** each pixel (x,y) in $I_{n}$ **do**

4:  $I_{f}(x,y)\leftarrow FractionalDerivative(I_{n},\alpha )$

5: **end** **for**

6: $G\leftarrow Tessellate(I_{f})$

7: Initialize CA states in G

8: **for** t=1 to T **do**

9:  **for** each cell *c* in G **do**

10:   UpdateState (c, MooreNeighborhood)

11:  **end** **for**

12: **end** **for**

13: $f_{CA}\leftarrow ExtractCAFeatures(G)$

14: $F\leftarrow CNN\_Forward(I_{f},\theta )$

15: $f_{CNN}\leftarrow GlobalAveragePooling(F)$

16: $f_{H}\leftarrow Concatenate(f_{CA},f_{CNN})$

17: $p\leftarrow Softmax(f_{H})$

18: y←arg⁡max(p)

19: **return** y

**Figure 2:**
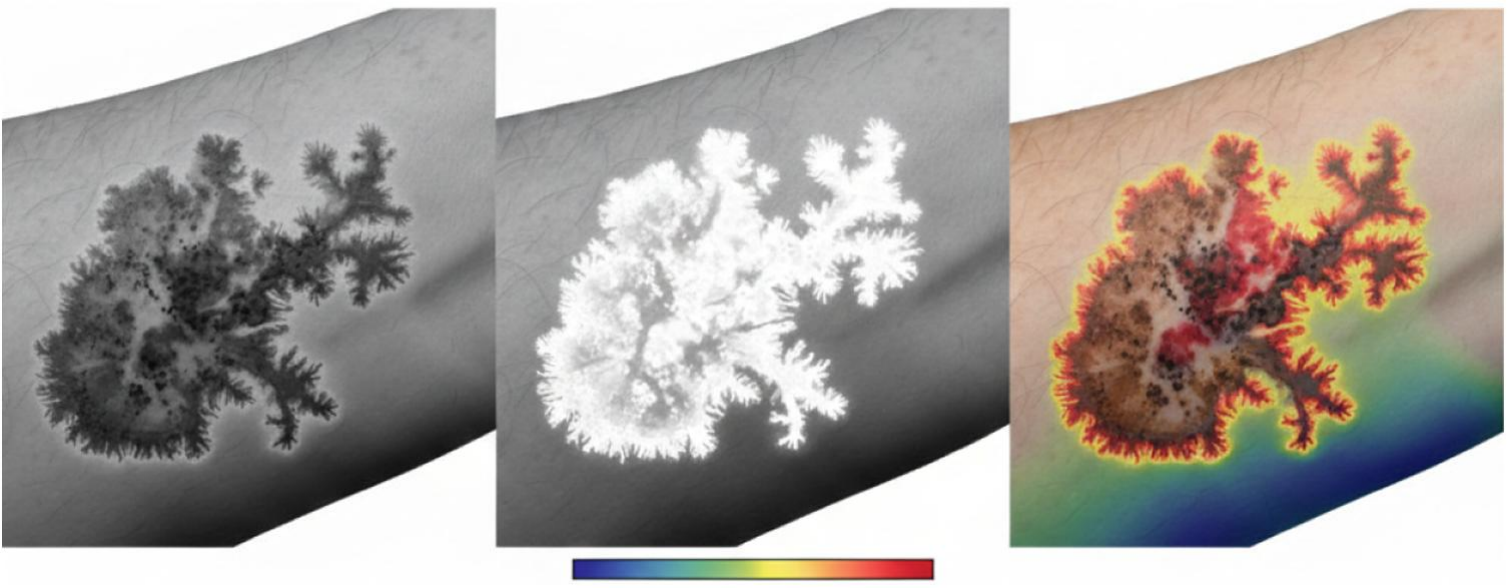
Heatmap-based visualization illustrating the explainability mechanism of the proposed framework. The figure shows the input image, the enhanced representation, and the corresponding attention heatmap highlighting the image regions that contribute most significantly to the classification decision.

A three-panel visualisation showcasing deep learning is shown in Figure 2. explainability for the investigation of skin lesions. The process starts with a greyscale input. highlighting texture data to a binary activation mask that delineates the area of curiosity, followed by a Grad-CAM heatmap. The heatmap’s red and yellow areas draw attention to abnormal characteristics such asymmetrical boundaries and pigment networks that participate in the classifying process. Areas of healthy skin are shown by blue areas. that the model appropriately ignores.

### 9.2 Skin type classification results

The system’s capacity to differentiate between dry, normal, and oily skin textures is assessed in the first stage. The overall effectiveness of various models for classifying skin types using common evaluation metrics is compiled in Table 10. The cellular automata model captures complementary structural information, whereas CNN and fractional-enhanced CNN models show good baseline performance. By successfully combining these elements, the hybrid framework improves F1-score, recall, accuracy, and precision. These findings imply that robust skin type categorisation is supported when deep learning is combined with fractional enhancement and cellular automata modelling.

**Table 10:** Overall skin type classification performance across different models.

Model	Accuracy	Precision	Recall	F1-Score
CNN	0.912	0.905	0.898	0.901
Cellular Automata (CA)	0.824	0.818	0.809	0.813
Fractional + CNN	0.918	0.913	0.906	0.909
**Hybrid (CNN + CA + Fractional CNN)**	**0.924**	**0.918**	**0.911**	**0.914**

Bold values are representing the performance of hybrid algorithms.

Table 10 illustrates that the hybrid framework proposed in this paper reached an accuracy of 0.924 vs. 0.912 for the baseline CNN model, implying a gain of approximately 1.2 percentage points.

Table 11 summarizes class-wise skin type classification for each modeling paradigm, to have a better insight into the performance of different models in classifying skin type of each class. Both CNN and CA models perform consistently across the three types of skin, i.e., Dry, normal and oily, each of them capturing distinct properties of each skin type. Fraction enhancement helps in distinguishing the class, by improving the representation of texture of dry and normal skin. Hybrid framework has equally well balanced precision, recall, specificity and F1-score across all the types of skin.

**Table 11:** Class-wise skin type performance metrics across different models.

Skin type	CNN				Cellular Automata (CA)			
	Precision	Recall	Specificity	F1-Score	Precision	Recall	Specificity	F1-Score
Dry skin	0.88	0.86	0.92	0.87	0.82	0.79	0.88	0.80
Normal skin	0.93	0.92	0.95	0.92	0.87	0.85	0.91	0.86
Oily skin	0.90	0.89	0.93	0.89	0.84	0.82	0.90	0.83

Skin type	Fractional + CNN				Hybrid (CNN + CA + Fractional CNN)			
	Precision	Recall	Specificity	F1-Score	Precision	Recall	Specificity	F1-Score
Dry skin	0.89	0.88	0.93	0.88	**0.90**	**0.89**	**0.93**	**0.89**
Normal skin	0.94	0.93	0.96	0.93	**0.94**	**0.95**	**0.96**	**0.94**
Oily skin	0.91	0.90	0.94	0.90	**0.91**	**0.90**	**0.94**	**0.90**

Bold values are representing the performance of hybrid algorithms.

**Figure 3:**
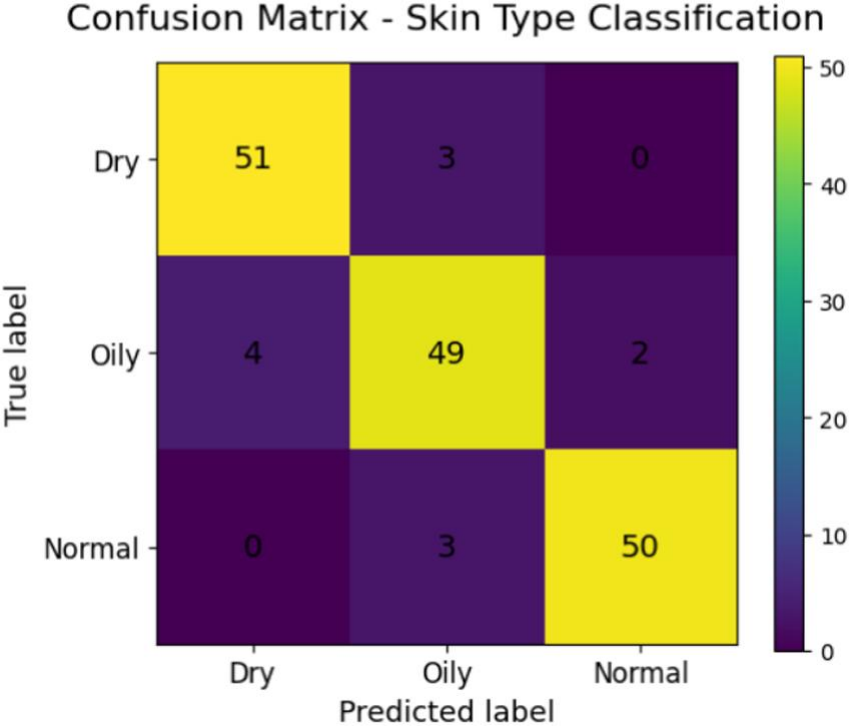
Confusion matrix for skin type classification.

The confusion matrix shown in Figure 3 is evidence for the reasoning above. The majority of predictions are found in the cells along the main diagonal, therefore confirming that most predictions are correct. Most misclassifications are of the form dry-oily or vice versa. This is expected medically since both skin types could display anomalies in pore grouping depending on atmospheric and hydrating conditions. In particular, it should be noted that normal skin is perfectly separated from both the other classes and that mid-level textures are learned by the hybrid model.

**Figure 4:**
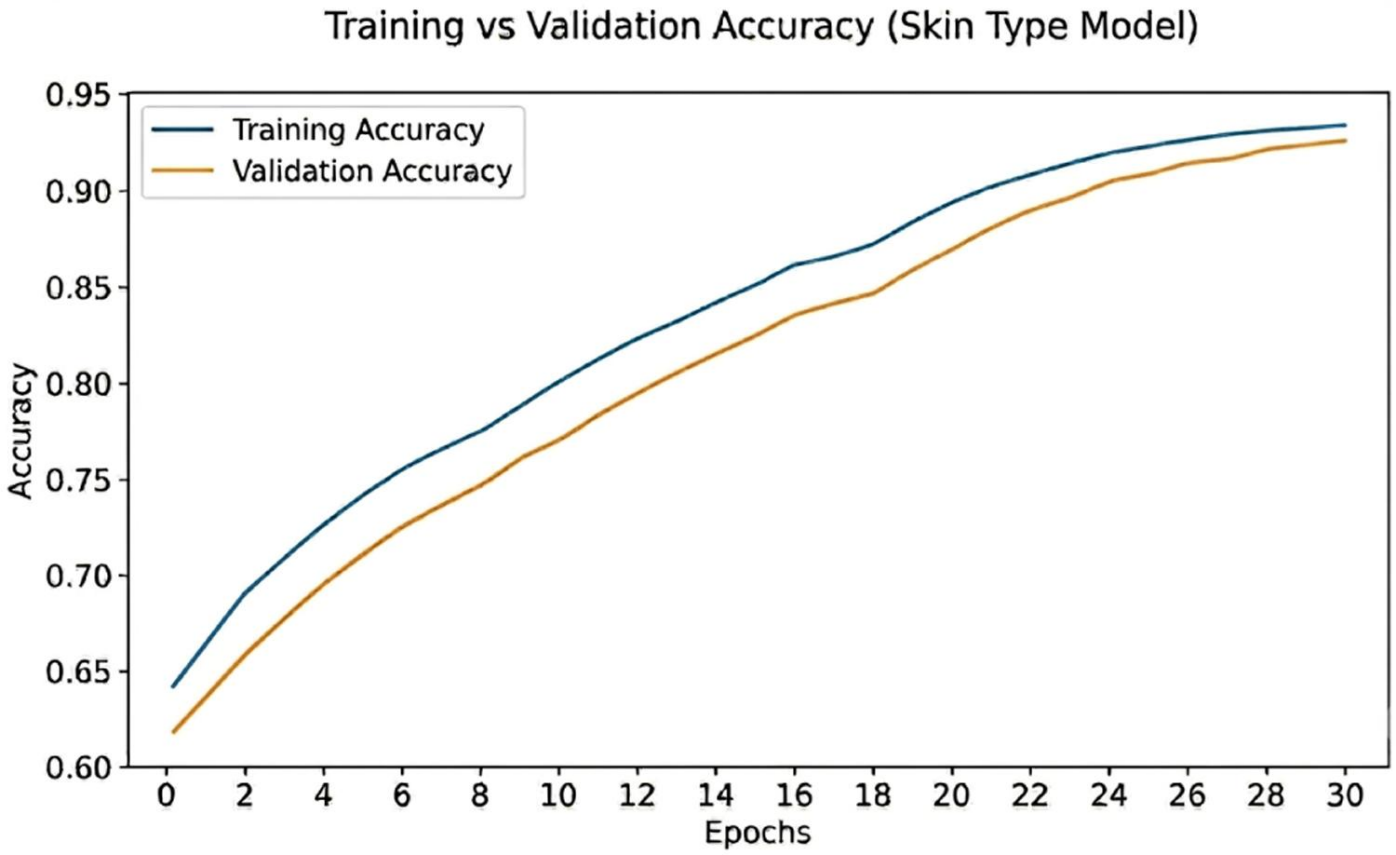
Training vs. validation accuracy for skin type model.

Figure 4 displays the training and validation accuracy curves over 30 epochs. Good generalisation is indicated by the convergence trend and the relatively small gap between the two curves throughout training. The model demonstrates effective learning behaviour and maintains stable performance across the training process. This consistent behaviour demonstrates that the integration of fractional-order enhancement and cellular automata-based texture modelling guides the CNN toward more robust and stable learning, resulting in improved classification performance.

### 9.3 Skin disease classification results

The second evaluation phase examines classification performance with respect to non-cancerous skin disorders. The overall performance of a different models for classification of skin diseases was summarized in Table 12 and compared using standard evaluation metrics. Both the CNN model and fractional-enhanced CNN model exhibit a solid baseline level of performance, while the cellular automata model captures complementary structural characteristics/attributes. The integration of fractional enhancement with the CNN results in increased precision and recall through improved representation of texture characteristics/attributes associated with disease pathology. Additionally, the hybrid framework further consolidates these strengths resulting in improved accuracy and balanced performance across all evaluation metrics.

**Table 12:** Overall skin disease classification performance across different models.

Model	Accuracy	Precision	Recall	F1-Score
CNN	0.912	0.904	0.897	0.899
Cellular Automata (CA)	0.836	0.829	0.821	0.825
Fractional + CNN	0.919	0.912	0.905	0.908
**Hybrid (CNN + CA + Fractional CNN)**	**0.928**	**0.921**	**0.914**	**0.917**

Bold values are representing the performance of hybrid algorithms.

Table 12 shows that the accuracy results of the proposed approach have been found to be 0.928 when compared with 0.912 of the base-line approach, where the accuracy was improved by about 1.6 percentage points.

Table 13 provides a detailed breakdown of how well each classification method had class-wise accuracy on the major skin diseases (e.g., acne, dermatitis, eczema, psoriasis and fungal infection) and how well the different methods did as a whole for these disease types. From these results we can see that CNN and fractional-enhanced CNNs had good prediction performance on all the major skin diseases; Another thing of importance to note is that the CA model was able to capture and detect additional structural patterns associated with these diseases. In terms of the hybrid framework’s overall prediction performance on these diseases, for all five of the listed skin disease types, there was a clear increase in prediction performance for all the listed skin disease types and improvements were particularly notable for acne and fungal infections. The results presented in this table suggest that our method demonstrates a good amount of generalizability for skin diseases.

**Table 13:** Class-wise skin disease classification accuracy across different models.

Disease	Accuracy			
	CNN	CA	Fractional+CNN	Hybrid
Acne	0.93	0.86	0.94	**0.96**
Dermatitis	0.88	0.82	0.90	**0.91**
Eczema	0.90	0.83	0.92	**0.93**
Psoriasis	0.86	0.80	0.88	**0.89**
Fungal infection	0.94	0.87	0.95	**0.97**

Bold values are representing the performance of hybrid algorithms.

**Figure 5:**
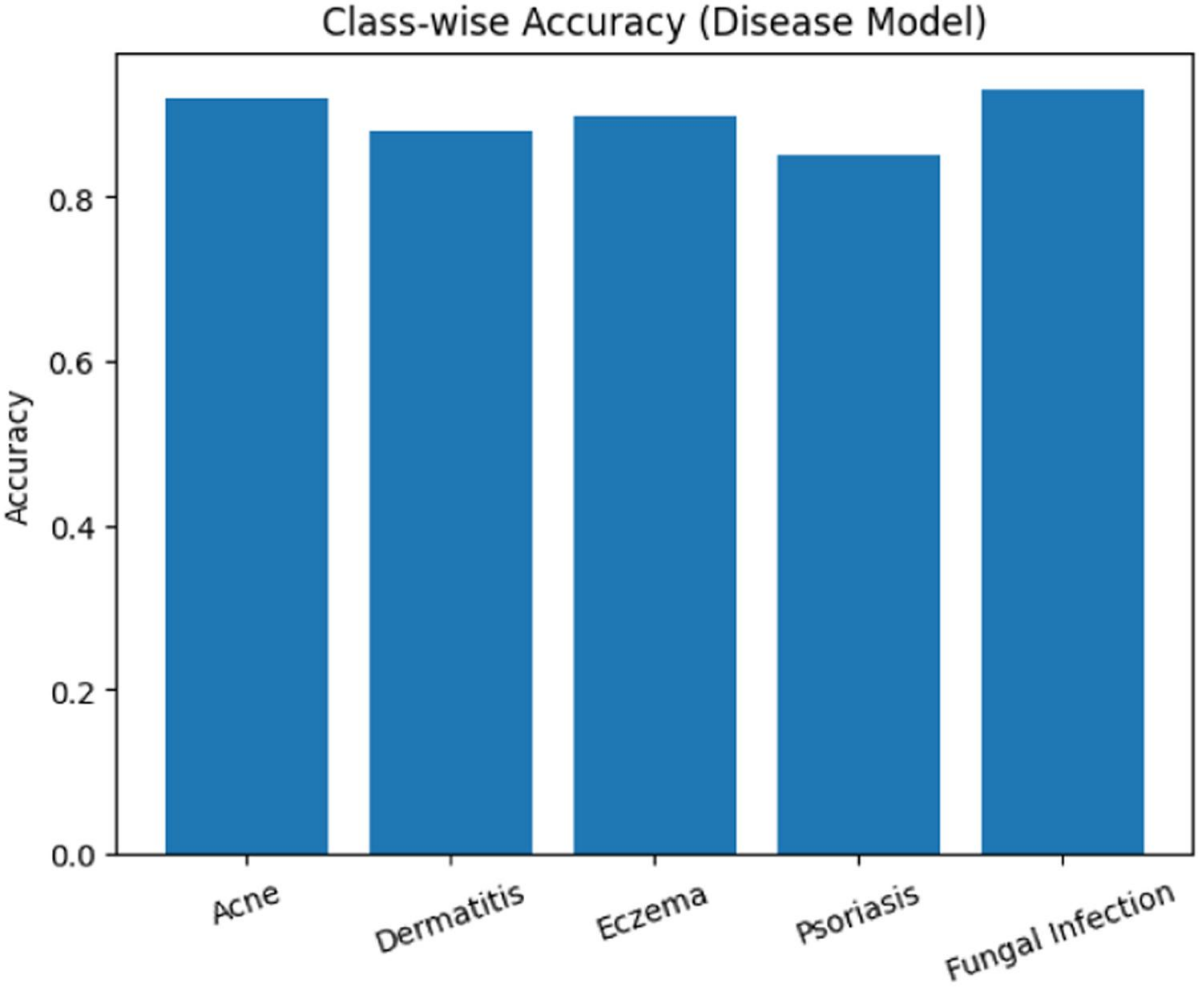
Class-wise accuracy for skin disease classification.

Through graphical depiction, the bar chart of Figure 5 supports the above observations by displaying comparative accuracy trends through multiple diagnoses. This type of graphical display will assist in quickly identifying areas of weakness, as well as allowing you to target certain areas for continued clinical refinement in the future.

**Figure 6:**
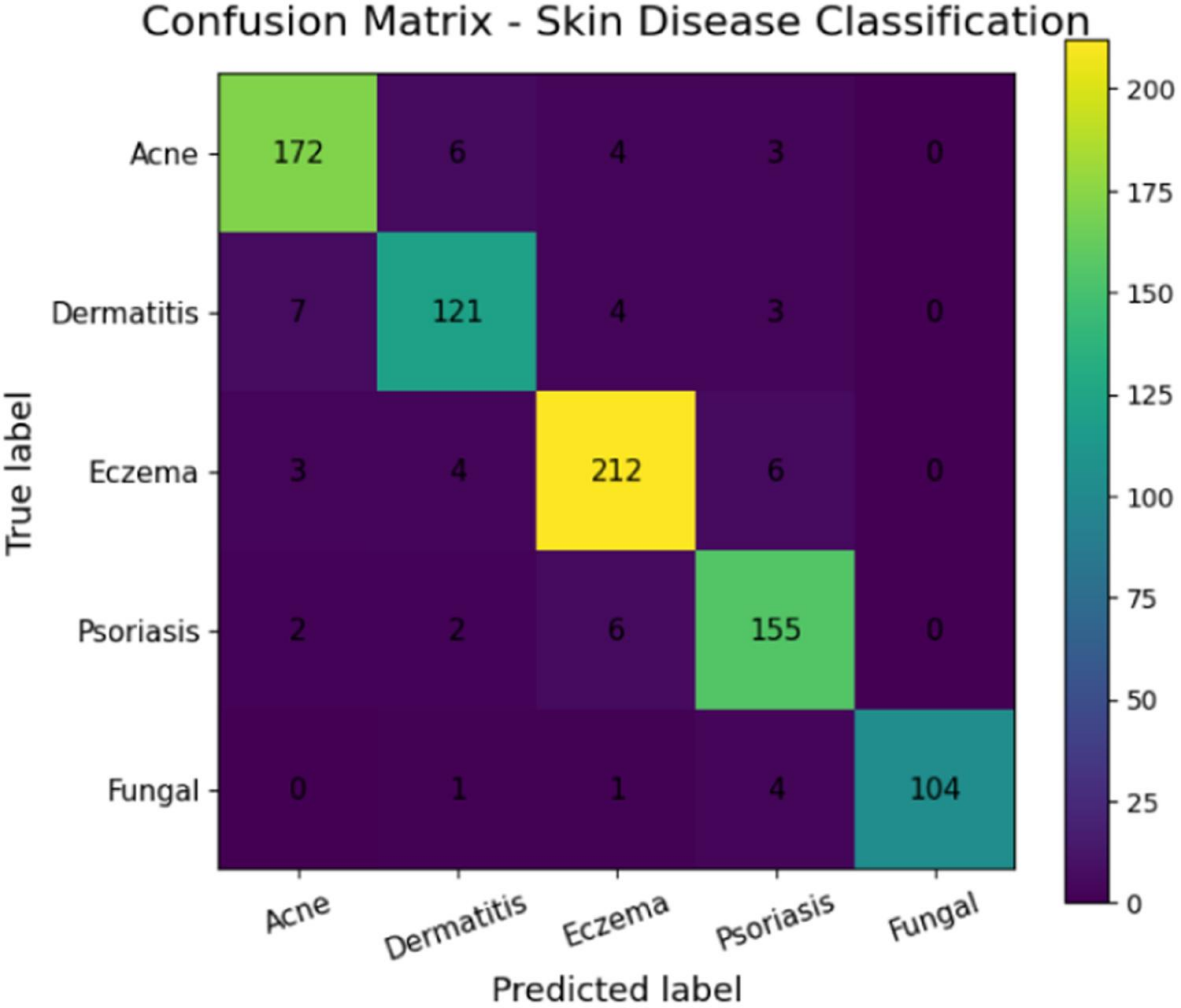
Confusion matrix for disease classification.

The classifications behavior is shown in the confusion matrix of Figure 6. The majority of the model’s predictions are diagonal, indicating that the model’s predictions generally match the true class for the majority of the data points. Instances that are misclassified tend to be located close to each other due to being morphologically similar, indicating that these errors are likely due to reasoned mistakes rather than random model confusion.

### 9.4 Comparative analysis

Each component’s part in two modes was analysed via performance on simpler configurations and then compared to hybrid implementation. Table 14 contains the performance of classification with/without math modeling used for skin type and skin disease tasks. The CNN model provides a strong deep learning baseline. The cellular automata and fractional enhancement introduce two complementary mathematical representations. Integrating fractional modeling with the CNN model shows an average of moderate improvements in performance on both tasks by combining all three methods into one hybrid composite framework produces similar gains in classification of skin type and skin disease.

**Table 14:** Performance comparison with and without mathematical modeling.

Model	Skin type accuracy	Skin disease accuracy
CNN	0.912	0.912
Cellular Automata (CA)	0.824	0.836
Fractional + CNN	0.918	0.919
**Hybrid (CNN + CA + Fractional CNN)**	**0.924**	**0.928**

Bold values are representing the performance of hybrid algorithms.

The proposed approach is compared with various studies available in the literature with respect to the comparison in Table 15. Studies conducted in previous works focused on deep learning frameworks, cellular automaton methods, fractional techniques, and hybrid learning methods for skin image analysis. The proposed approach combines the concepts of convolutional neural networks, cellular automata, and fractional order differentiation into one approach for both skin type recognition and skin disease classification tasks.

**Table 15:** Comparison of the proposed framework with existing studies.

Study	Method	Main contribution
Esteva et al. [2]	Deep Neural Networks	Dermatologist-level skin cancer classification.
Abbasi et al. [6]	Deep CNN	Multi-class skin cancer classification.
Hosny and Kassem [5]	Residual CNN	Skin lesion classification using refined residual networks.
Alruwaili and Mohamed [7]	EfficientNet + ResNet	Multiclass skin disease classification using integrated deep learning models.
Luna-Benoso et al. [10]	Cellular Automata	Cellular automata-based melanoma detection in dermoscopic images.
Zhang et al. [15]	Fractional Enhancement	Fractional differentiation-based enhancement for melanoma image analysis.
Jamal et al. [24]	TopoResNet	Hybrid deep learning architecture incorporating topological features for skin lesion classification.
Jabber et al. [26]	K-means + LAB + Deep Learning	Automated skin lesion diagnosis using segmentation and deep learning techniques.
Proposed Work	CNN + Cellular Automata + Fractional Derivatives	Unified framework for skin type identification and skin disease classification with interpretable texture modelling and hybrid feature representation.

The purpose of applying noise suppression is to remove any extraneous effects created by lighting and sensor inaccuracies that affect the quality of skin images. Table 16 displays how noise suppression has affected the accuracy and F1-score of multiple modelling techniques for skin image classification. Inclusion of noise suppression not only leads to better accuracy and F1-scores but also suggests an increase in the distinctiveness of the surface components of the images used as input to the different modelling techniques. Both fractional enhanced models, as well as the hybrid model benefited from noise suppression. Impressive texture detail allows those two types of models to produce results which are deemed more reliable. Therefore, it is evident that applying preprocessing techniques has a positive impact upon improving overall model performance.

**Table 16:** Performance comparison with and without noise removal across different models.

Model	Configuration	Accuracy	F1-Score
CNN	Without Noise Removal	0.872	0.868
	With Noise Removal	**0.889**	**0.885**
Cellular Automata (CA)	Without Noise Removal	0.804	0.798
	With Noise Removal	**0.824**	**0.818**
Fractional + CNN	Without Noise Removal	0.905	0.900
	With Noise Removal	**0.918**	**0.914**
**Hybrid (CNN + CA + Fractional CNN)**	Without Noise Removal	0.912	0.907
	With Noise Removal	**0.924**	**0.914**

Bold values are representing the performance of hybrid algorithms.

The use of data augmentation enhances model generalizability by increasing training data variability. The results of Table 17 show how well each classification performance method compares with those of respective models that did not employ data augmentation. All models show an improvement in both accuracy and F1 score, which indicates better model generalization; fractional-enhanced and hybrid configurations appear to exhibit the greatest benefit due to their ability to create sample diversity through the addition of augmented data to provide a more robust learning environment. These results support the effectiveness of data augmentation as a strategy for improving model performance.

**Table 17:** Performance comparison with and without data augmentation across different models.

Model	Configuration	Accuracy	F1-Score
CNN	Without Data Augmentation	0.884	0.879
	With Data Augmentation	**0.901**	**0.897**
Cellular Automata (CA)	Without Data Augmentation	0.812	0.806
	With Data Augmentation	**0.824**	**0.818**
Fractional + CNN	Without Data Augmentation	0.905	0.900
	With Data Augmentation	**0.918**	**0.914**
**Hybrid (CNN + CA + Fractional CNN)**	Without Data Augmentation	0.912	0.907
	With Data Augmentation	**0.924**	**0.914**

Bold values are representing the performance of hybrid algorithms.

The results from integer-order and fractional-order enhancement methods combined with the use of each of the models in this study can be found in Table 18. Integer-order operators produce a uniform, steady enhancement baseline, as opposed to fractional-order enhancements which provide an enhanced level of texture and edge preservation. In all three of the models studied, the use of fractional-order enhancement correlated with statistically significant increases in both the accuracy and F1 scores. This indicates that fractional-order enhancement is an effective complementary preprocessing technique for performing skin image analysis.

**Table 18:** Performance comparison of integer-order and fractional-order enhancement across different models.

Model	Enhancement Method	Accuracy	F1-Score
CNN	Integer-Order Operators	0.893	0.889
	Fractional-Order Enhancement	**0.912**	**0.908**
Cellular Automata (CA)	Integer-Order Operators	0.812	0.806
	Fractional-Order Enhancement	**0.824**	**0.818**
Fractional + CNN	Integer-Order Operators	0.905	0.900
	Fractional-Order Enhancement	**0.918**	**0.914**
**Hybrid (CNN + CA + Fractional CNN)**	Integer-Order Operators	0.912	0.907
	Fractional-Order Enhancement	**0.924**	**0.914**

Bold values are representing the performance of hybrid algorithms.

The effectiveness of different formulations of fractional derivatives in improving texture has been assessed. Table 19 shows how the caps of the Grüwald-Letnikov fractional derivative and the Caputo fractional derivative have an effect on the classification rates when different modelling techniques are used. Therefore, regardless of the model used, both fractional derivatives enhanced the textures through the capturing of fractional order properties of skin based images. The Caputo fractional derivative performed slightly better across all models (e.g., accuracy and F1-score) which indicates a smoother and more stable texture refinement process. The results support the conclusion that the type of fractional derivative plays a part in determining enhancement effectiveness across several different modelling types.

**Table 19:** Comparison between Grünwald–Letnikov and Caputo fractional derivatives across different models.

Model	Fractional derivative	Accuracy	F1-Score
CNN	Grünwald–Letnikov	0.908	0.904
	Caputo	**0.914**	**0.910**
Cellular Automata (CA)	Grünwald–Letnikov	0.820	0.815
	Caputo	**0.824**	**0.818**
Fractional + CNN	Grünwald–Letnikov	0.915	0.910
	Caputo	**0.918**	**0.914**
**Hybrid (CNN + CA + Fractional CNN)**	Grünwald–Letnikov	0.921	0.916
	Caputo	**0.924**	**0.919**

Bold values are representing the performance of hybrid algorithms.

Using tessellation-based cellular automata (CA) modeling to obtain neighborhood-level texture evolution is one way to capture this information. The effects of adding CA features to the classification framework are summarized in Table 20. The models that have cellular automata improvements consistently achieve better accuracy and F1 scores, demonstrating that modeling texture at the neighborhood level is advantageous. Furthermore, fractional-enhanced and hybrid configurations reap significant rewards from incorporating cellular automata by allowing them to better model their correlations with each other across space. Overall, these findings show how cellular automata can enhance the classification performance of deep learning models through a complementary relationship between the two approaches.

**Table 20:** Performance comparison with and without cellular automata modeling across different models.

Model	CA configuration	Accuracy	F1-Score
CNN	Without Cellular Automata	0.912	0.908
	With Cellular Automata	**0.921**	**0.916**
Cellular Automata (CA)	Without Cellular Automata	0.824	0.818
	With Cellular Automata	**0.836**	**0.825**
Fractional + CNN	Without Cellular Automata	0.918	0.914
	With Cellular Automata	**0.924**	**0.919**
**Hybrid (CNN + CA + Fractional CNN)**	Without Cellular Automata	0.924	0.914
	With Cellular Automata	**0.932**	**0.919**

Bold values are representing the performance of hybrid algorithms.

**Figure 7:**
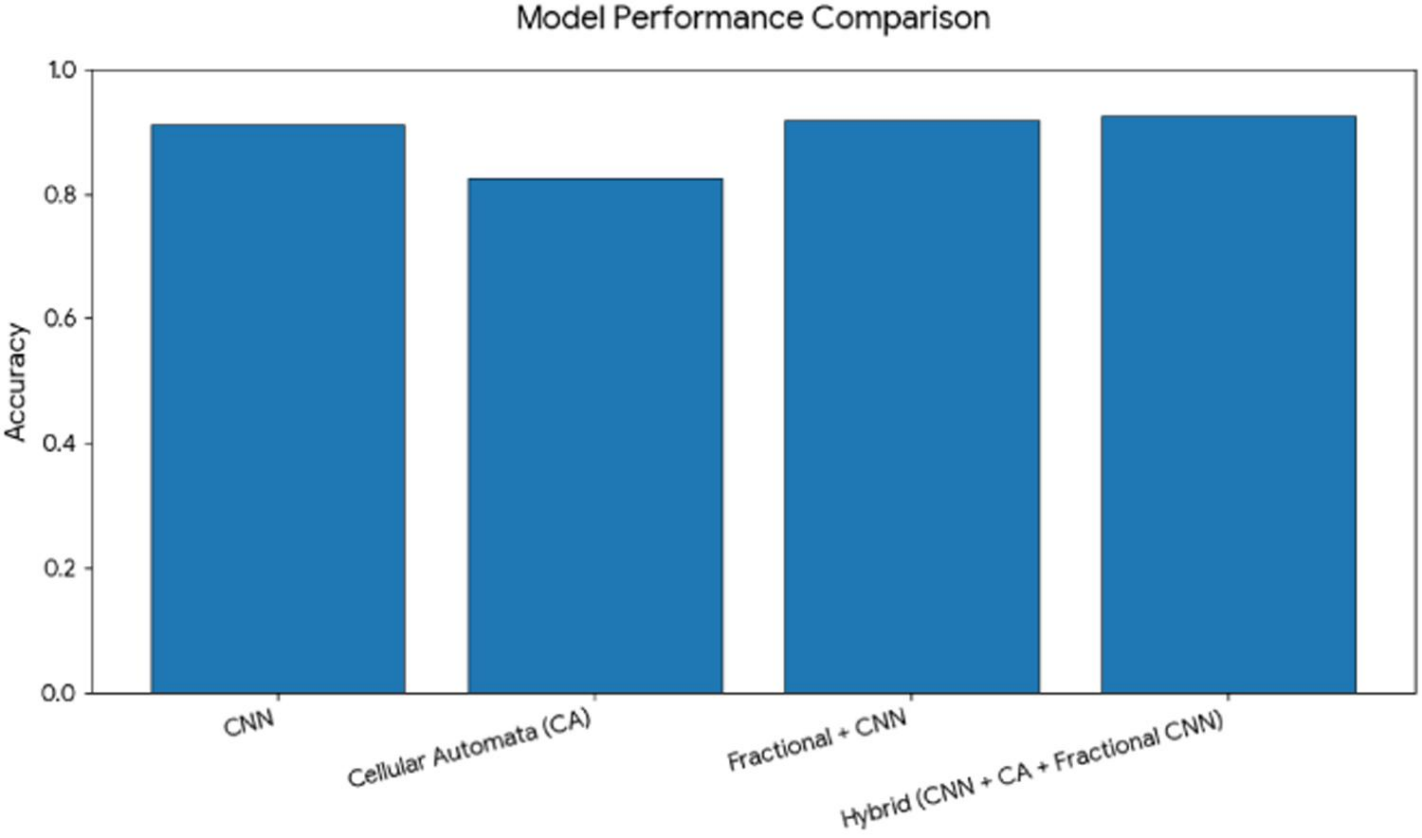
Performance comparison of baseline and proposed models.

Figure 7 visually highlights improvement margins, clearly showing that hybrid modeling consistently remains higher than baseline systems.

**Figure 8:**
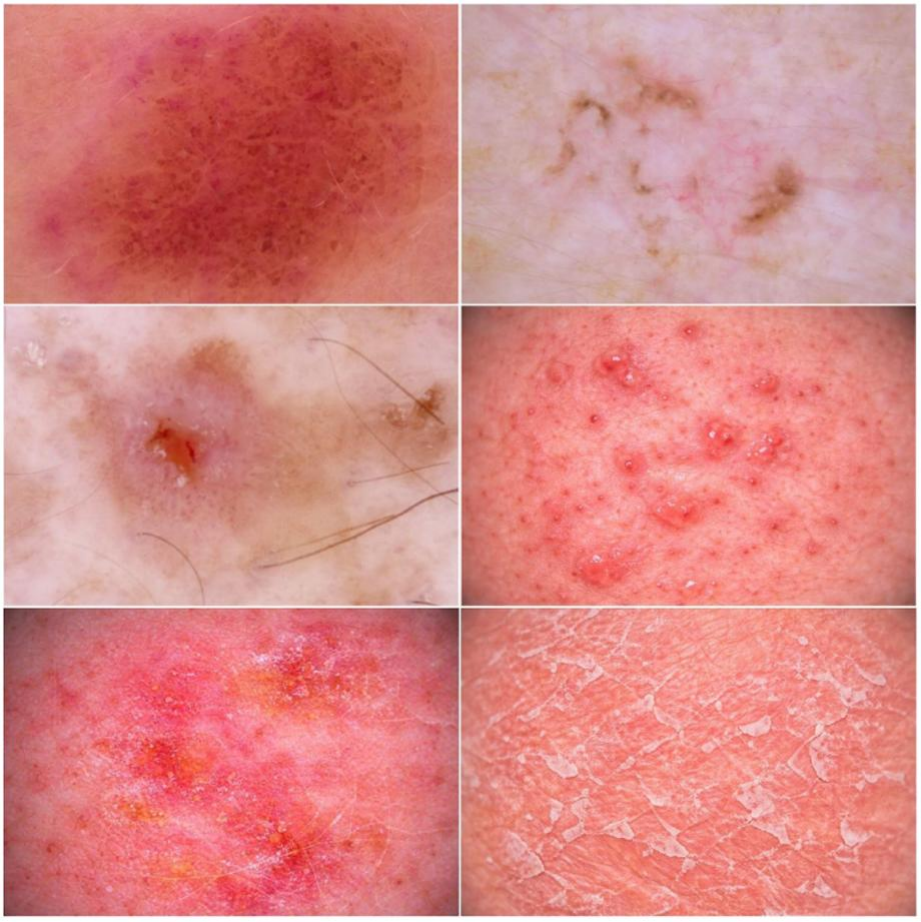
Sample images from the dataset showing variations in skin types and dermatological conditions.

Figure 8 shows some representative sample images selected from the dataset used for this study, where the difference in human skin types and skin diseases can be visualized according to the differences in appearances of skin surfaces. It is clearly demonstrated that the diversity of texture, color distribution, and structure of skin surfaces makes it important to address the robustness of the proposed framework for skin type and skin disease classification.

**Figure 9:**
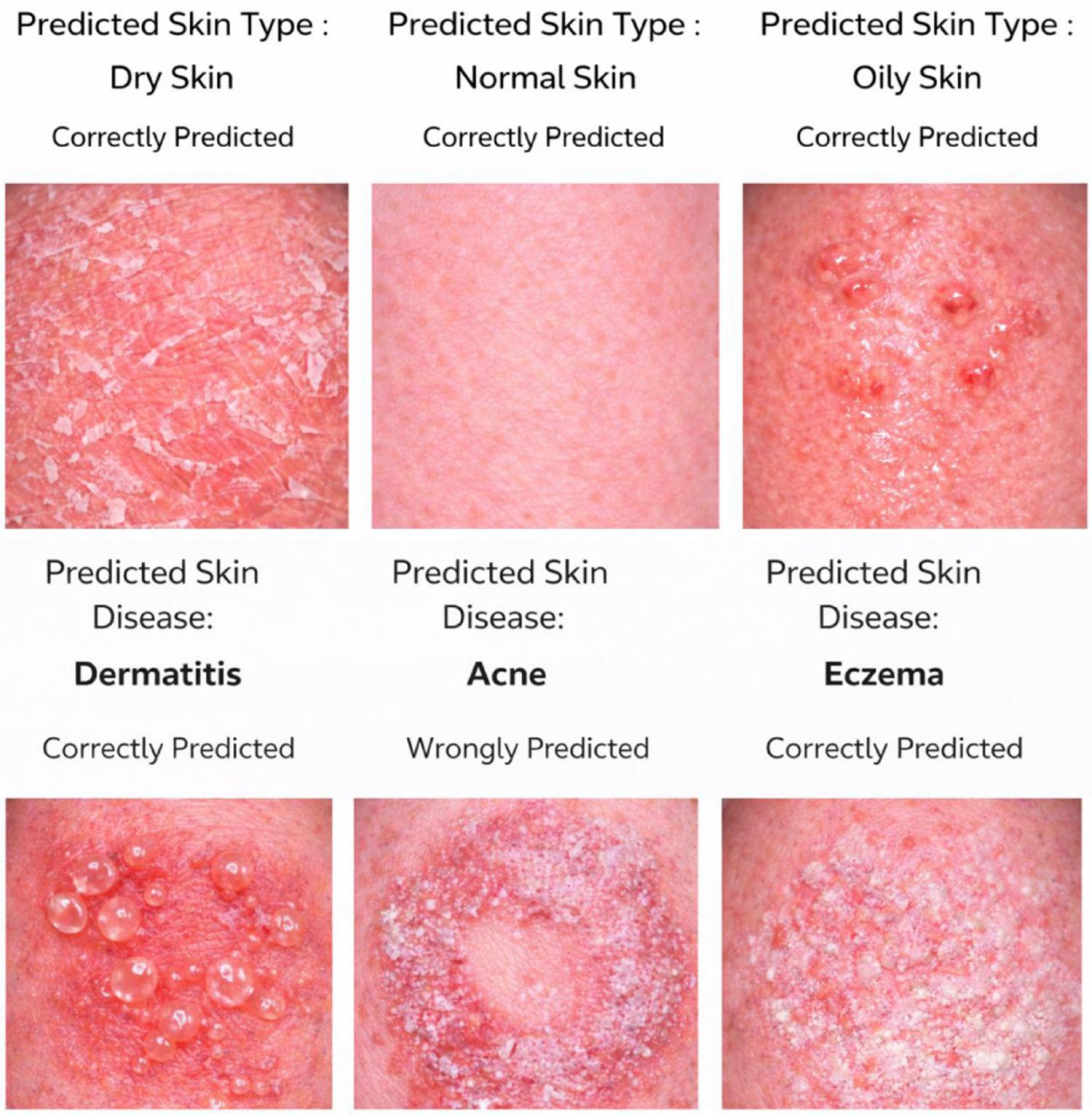
Prediction results for skin type and skin disease classification, showing both correct and incorrect model predictions.

Figure 9 presents prediction results from the system proposed for skin type and skin disease classification. It visually presents both correct and incorrect predictions,indicating how the model responds to different surface texture characteristics.

### 9.5 Failure analysis

#### 9.5.1 Illumination and image quality variations

Misclassification is also caused by uneven lighting, shadowing, and low contrast in the images of skin. Because the fine-textured patterns of the skin and the boundary of a lesion cannot be visually detected well when there is poor lighting, fractional enhancement and attention mechanisms will be less effective in identifying the location of the lesion. Therefore, when skin images are captured in uncontrolled lighting environments, the prediction uncertainty will be greater than if there were control over these variables.

#### 9.5.2 Similar visual characteristics between skin conditions

Psoriasis and Dermatitis are two skin diseases where the texture of the skin is similar, and the colors will overlap as well, making it difficult to differentiate from one another when classifying the diseases, even when using Feature Extraction Technology. This leads to incorrect classifications because at times the model misreads the scaling and redness.

#### 9.5.3 Small or diffused lesion regions

Models may not provide enough attention to the abnormal area if the affected skin area is a very small portion within the image, or diffusely located across the surface. This problem is most apparent in early-stage disease images, where the boundaries of a lesion can be difficult to delineate.

#### 9.5.4 Background interference and occlusions

Background features (e.g., hair) have the potential to create distractions that will influence an individual’s ability to successfully localise and classify features because these features will introduce noise to the learning process and possibly distract attention mechanisms.

To summarize, the findings obtained from this study provided strong evidence for validating the proposed system’s design. The use of fractional derivatives improved the clarity of skin texture and increased the model’s ability to detect subtle changes. The introduction of cellular automata provided a neighbourhood based on interpretable learning that demonstrates the way skin texture changes, as opposed to considering each pixel alone. The use of deep CNNs provided additional features that provide higher levels of perception for use in identifying skin diseases. The aforementioned methods when used together have resulted in a significant reduction of ambiguity, improved classification accuracy and therefore increased reliability of decision making. Hence the hybrid framework has provided a reasonable means of bridging the gap between accuracy and interpretability thus indicating an exciting future direction for developing dermatological AI solutions.

## Data Availability

The original contributions presented in the study are included in the article/Supplementary Material, further inquiries can be directed to the corresponding author/s.
